# Early pregnancy detection by female community health volunteers in Nepal facilitated referral for appropriate reproductive health services

**DOI:** 10.9745/GHSP-D-12-00026

**Published:** 2013-09-16

**Authors:** Kathryn Andersen, Anuja Singh, Meena Kumari Shrestha, Mukta Shah, Erin Pearson, Leila Hessini

**Affiliations:** a Ipas, Chapel Hill, NC, USA; b Ipas, Kathmandu, Nepal

## Abstract

**Background:**

Female community health volunteers (FCHVs) are a possible entry point for Nepali women to access timely reproductive health services at the village level. This evaluation assessed the success of a pilot program that trained FCHVs in early pregnancy detection using urine pregnancy tests (UPTs), counseling, and referral to appropriate antenatal, safe abortion, or family planning services.

**Methods:**

Between July 2008 and June 2009, the program trained 1,683 FCHVs from 6 districts on how to provide UPTs and appropriate counseling and referral; 1,492 FCHVs (89%) provided follow-up data on the number of clients served and the type of services provided. In addition, the program conducted in-depth interviews with selected FCHVs and other reproductive health service providers on their perceptions of the program.

**Results:**

Of the FCHVs with follow-up data, 80% reported providing UPTs to women in the 8-month follow-up period. In total, they conducted 4,598 UPTs, with a mean number of 3.1 tests per FCHV. Among the women with a negative pregnancy test (47%), FCHVs provided 24% of them with oral contraceptive pills and 20% with condoms; referred 10% for other contraceptive services; and provided contraceptive counseling only to 46%. Among the women with positive pregnancy tests (53%), FCHVs referred 68% for antenatal care and 32% for safe abortion services.

**Conclusions:**

Providing FCHVs with the skills and supplies required for early pregnancy detection allowed them to make referrals for appropriate reproductive health services. Results of this evaluation suggest that community health workers such as FCHVs are a promising channel for early pregnancy detection and referral. As the intervention is scaled up, the focus should be on ensuring service availability and awareness of available services, UPT supply, and creating viable options for record keeping.

## Abstract (precis)

Trained female community health volunteers provided low-cost urine pregnancy tests in their communities, leading to counseling and appropriate referrals for antenatal care, family planning, or comprehensive abortion care.

## BACKGROUND

Timely identification of pregnancy is important for initiating antenatal care (ANC) among women with wanted pregnancies and for seeking abortion services among women with unwanted pregnancies. Although abortion is a safe procedure, the medical risk is greater at higher gestational ages.^1^ In addition, early identification of an unwanted pregnancy is important so that women can access abortion services before they exceed the legally allowed gestational limit.

Lack of certainty about pregnancy status can cause delays in seeking ANC or abortion. For example, a study of ANC clients in South Africa found that women waited up to 5 months gestation before feeling confident that they were pregnant and seeking care, and women expressed difficulties with identifying pregnancy when menses are typically irregular.^2^

Women who are unsure whether they are pregnant sometimes delay seeking antenatal care or abortion services.

Urine pregnancy testing has been shown to decrease the gestational age at which women seek antenatal care and abortion services.^3^^–^^4^ A study in South Africa showed that women who used urine pregnancy tests (UPTs) presented for ANC services 3.6 weeks earlier, and abortion clients 1.4 weeks earlier, than women who did not use UPTs.^3^ Another study from South Africa found that introducing a pregnancy confirmation clinic using UPTs within existing ANC clinics decreased the mean gestational age at which women began ANC.^4^ Although this study used facility-based distribution of pregnancy tests, community-based provision of pregnancy tests may further increase access to reproductive health services, especially in rural areas.

In Nepal, ANC and comprehensive abortion care (CAC) services are available in government facilities, but women often delay seeking services due to geographical barriers and social barriers against traveling alone to health facilities.^5^ In addition, husbands and mothers-in-law in Nepal often act as gatekeepers who prevent women from accessing health services.^6^

The World Health Organization (WHO) recommends at least 4 ANC visits, starting as early as possible in the first trimester.^7^ But in Nepal, the median gestational age at the first ANC visit is 3.7 months, indicating that many women are not seeking services until their second trimester.^5^

Under Nepal's current abortion law, implemented in 2004, women have the legal right to an abortion up to 12 weeks of gestation for any indication; up to 18 weeks of gestation in the case of rape or incest; and at any time during pregnancy, if the mental or physical health or life of the pregnant woman is at risk or if the fetus is deformed and incompatible with life, with the advice of a medical practitioner.^8^ Access to services in the first trimester is important for both ANC and CAC clients.

Increasing the availability of urine pregnancy testing in Nepal may be one way to enable women to identify pregnancy earlier and seek more timely ANC or CAC services. Currently, UPTs are available in Nepal through medical shops, but access is limited in remote areas and cost can be a barrier. The cost of UPTs in medical shops outside the capital Kathmandu ranges from Rs. 50–60 (US$0.58–0.70), which is almost half the daily income for the majority of Nepal's population (78%) living below US$2 per day.^9^

One promising approach, started by Nepal's Ministry of Health and Population in 1988, uses community-based volunteers, known as female community health volunteers (FCHVs), to reach women in remote areas and link them with available services. This network, currently comprising more than 48,000 local female volunteers,^10^ is a trusted source of maternal and child health information and serves as the key referral link between communities and health services.

FCHVs are selected by the Mothers Group at the village development committee (VDC) level, and each ward within the VDC typically has one FCHV. Criteria for selection include membership in the VDC, literacy, and age between 25 and 45 years, but the actual characteristics of FCHVs vary. A study found that the median age for FCHVs was 38 years and that 4% of FCHVs were over age 60.^11^ The study also found that only 62% of FCHVs were literate, but FCHVs were better educated than the general population of rural women of the same age.^11^ Literacy levels did not affect the quality of services provided by FCHVs.^11^

Once selected, FCHVs attend an 18-day training on basic maternal and child health information; training on additional topics is conducted as needed. Services provided by FCHVs once they return to their communities include^11^:

- Holding monthly meetings on health issues with the Mothers Group in their villages
- Visiting households to advise on maternal and newborn care and immunizations and to distribute condoms and oral contraceptive pills to existing users
- Iron/folate distribution
- Postpartum vitamin A distribution
- Treatment of children with diarrhea using oral rehydration solution
- First aid
- Community-based pneumonia treatment in some districts

FCHVs are not permitted by law to provide injections or any medical procedure. FCHVs do not receive compensation for their work, but they do get reimbursed for travel expenses when attending trainings and they receive money to purchase tea and snacks for their monthly Mothers Group meetings. FCHVs work, on average, 5.1 hours each week, and annual turnover is low (4%).^11^

Since inception of the program, FCHVs have provided referrals for ANC and family planning services. After liberalization of the abortion law in Nepal, Ipas, an international NGO focused on preventing unsafe abortion, began training FCHVs to refer women for safe abortion services in communities where services were available, initially based on women's suspicion of pregnancy.

In 2008, Ipas conducted a feasibility study in Bhaktapur District to test the potential of FCHVs to use urine pregnancy testing to link Nepali women to timely reproductive health services. A total of 230 FCHVs were trained to identify early pregnancy using UPTs and to make appropriate referrals to ANC or CAC services for women with positive pregnancy tests and to family planning services for women with negative pregnancy tests. After implementation of this intervention, use of antenatal care, family planning, and safe abortion services improved in the target communities.^12^

Based on this success, Ipas and its partners conducted a similar but larger-scale pilot program covering FCHVs from 6 districts in Nepal (Chitwan, Dhading, Jhapa, Kailali, Surkhet, and Tanahu). The purpose of this paper is to present this innovative approach, which relies on volunteers to link rural women to reproductive health services, and to present initial evaluation results from the 6-district pilot.

## INTERVENTION DESCRIPTION

**Figure 1.**
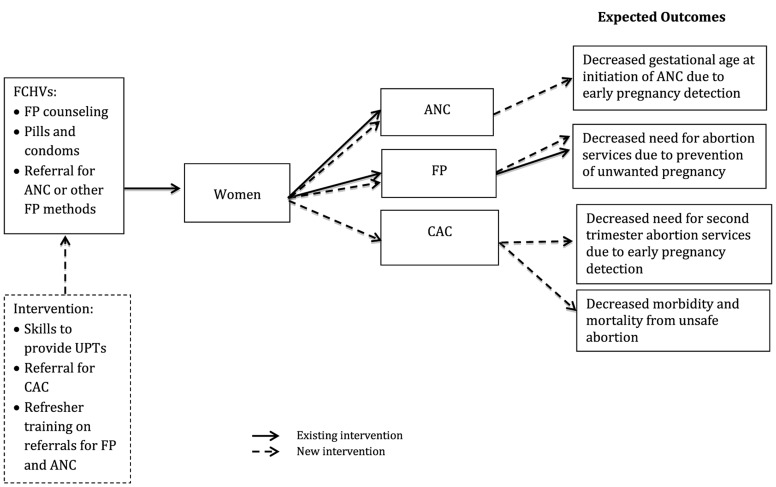
Conceptual Model for the “Early Pregnancy Detection and Reproductive Health Counseling Program” Abbreviations: ANC, antenatal care; CAC, comprehensive abortion care; FCHVs, female community health volunteers; FP, family planning; UPT, urine pregnancy test.

The “Early Pregnancy Detection and Reproductive Health Counseling Program” trained FCHVs on proper use of UPTs and referral for safe abortion and ANC, as well as additional training on family planning, to increase access to reproductive health services through early pregnancy detection at the community level. The focus of the intervention was on training FCHVs to provide UPTs and referral for safe abortion services, if the woman desired, in addition to her typical duties of family planning counseling, provision of condoms and pills to existing users, and referral for ANC or other contraceptive methods, such as sterilization, injectables, and intrauterine devices (IUDs). Early pregnancy detection is hypothesized to decrease the gestational age at which: (a) women with wanted pregnancies initiate ANC and (b) women with unwanted pregnancies seek safe abortion services (Figure 1). In addition, the intervention supplied FCHVs with knowledge of where to seek safe abortion services, hypothesized to decrease morbidity and mortality associated with unsafe abortion.

In conjunction with the Ministry of Health, Ipas conducted training of trainer (TOT) workshops with medical officers in-charge and providers working at the primary health care center (PHCC), health post, and sub-health post levels. Between January and June 2009, the trainers conducted a series of 2-day workshops for FCHVs at each selected VDC in the 6 districts. Invited FCHVs were from districts where Ipas was also implementing a program to provide medical abortion (MA) as an alternative to surgical abortion methods. All FCHVs working in the catchment area of the facilities providing MA and manual vacuum aspiration (MVA) were trained. One FCHV training was conducted in each VDC, and each training included 9–25 FCHVs, depending on the number working in the area. A total of 1,683 FCHVs were trained.

The training workshops used a variety of interactive methods and formats to promote adult learning, including brainstorming, group discussion, question and answer sessions, mini-lectures, and demonstrations. Topics included:

- How to perform UPTs, practicing on both pregnant and non-pregnant urine specimens
- Informing women in their communities about the availability of UPTs through the existing monthly Mothers Group meetings
- Reinforcement of previous training on contraception and referral for ANC and additional family planning services
- New content on safe abortion, including conditions under which abortion is legal in Nepal, consequences of unsafe abortion, names and locations of certified CAC sites in the FCHVs' districts, and costs associated with CAC services
- Introduction to specially designed information, education, and communication (IEC) materials for use with low-literacy women
- Use of pictorial referral cards designed for use with women with low literacy levels (one section of the referral card included the referral information, which was given to the women, while the FCHVs kept the other section for record-keeping purposes)

On completion of the training, the program provided each FCHV with 15 UPT kits for use in her community; 5 kits were free and the FCHV paid Rs. 10 (US$0.12) per test for the other 10 kits. At the training, the FCHVs agreed that they would charge community members up to Rs. 27 (US$0.32) per test, providing them with a small financial incentive to provide this service. FCHVs charged on a sliding scale depending on what the woman could afford.

The program instructed FCHVs to purchase additional UPT kits as needed through local medicine shops. Most chemist shops outside Kathmandu charge Rs. 50–60 for UPTs. For these replacement UPTs, FCHVs agreed to charge women up to Rs. 50, again on a sliding scale, according to what women could afford.

FCHVs informed women in the community about availability of UPTs primarily through Mothers Group meetings. During the meetings, the FCHVs informed women that if they suspected pregnancy, they could come to the FCHVs for a test. In addition, FCHVs informed women about the availability of UPTs informally when the women sought other information from the FCHVs.

## METHODS

Stakeholders from the Family Health Division (Ministry of Health), the FCHV program, Ipas, and the Group for Technical Assistance (GTA)—a Nepalese nonprofit with expertise in community and human resource development—planned the evaluation to assess performance of the trained FCHVs in providing community-based early pregnancy detection and reproductive health counseling and referral services. In addition, we assessed the usefulness of the IEC materials in supporting the FCHVs' reproductive health counseling for women, especially for those seeking CAC services. The Allendale Investigational Review Board reviewed and approved the evaluation protocol and granted a waiver of informed consent as the study presented no more than minimal risk to participants.

### Participants

Of the 1,683 trained FCHVs, 1,492 (89%) participated in a 1-day follow-up review meeting conducted at the VDC level to assess their progress and performance. The review meetings took place approximately 8 months after the initial 2-day training sessions (between November 2009 and March 2010). During these meetings, all previously trained FCHVs provided self-reported data using record sheets, and a subset of FCHVs participated in semi-structured interviews about the IEC materials and their perceived impact of the program. These data were supplemented by interviews conducted with 9 government and NGO reproductive health service providers.

### Data Sources and Procedures

#### FCHV Record Sheets

We developed a structured format to collect information from the FCHVs on:

- Number of pregnancy tests they performed since the training
- Results of the pregnancy tests
- Reasons for not using the pregnancy test kits, if applicable
- Whether the FCHVs provided family planning counseling and/or had distributed condoms or oral contraceptive pills to women in their communities
- Number of women the FCHVs referred for additional family planning services, ANC, and CAC services

The FCHVs maintained records throughout the study period by retaining half of each referral card that they provided to women in their community; they brought these referral cards to the review meeting to help complete the structured data collection tool.

#### Semi-Structured Interviews With FCHVs

We also designed a semi-structured interview guide to supplement and contextualize the quantitative data from the record sheets. Each review meeting included approximately 9 FCHVs; we randomly selected 3–5 of these FCHVs for an interview to collect information on:

- How the FCHVs were using information from the training in their communities
- How many cases they referred for services since the training and for what types of services
- Their perceptions of community benefits resulting from their training
- The utility of the IEC materials (specifically, whether they used the flip chart in Mothers Group meetings; whether the messages and language used in the flip chart were clear and understandable; and how they used the pregnancy test kit materials, which consisted of a bag, brochure, key ring, and referral card)

#### Semi-Structured Interviews With Reproductive Health Care Providers

We also collected data from 9 government and NGO service providers on their perceptions of the program's effectiveness, using a semi-structured interview guide. Specifically, we asked providers about their perceptions of the program's effect on FCHV referrals and their thoughts on other ways to increase women's access to reproductive health services.

### Analysis

Quantitative data were entered into EpiInfo and checked for consistency. We report descriptive statistics in this paper. We did not collect data from interviews systematically. Some of the interviews were videotaped, transcribed, and translated into English. However, others were not recorded and only had interviewer notes. We reviewed the transcripts and interviewer notes for common themes and selected illustrative quotes.

## RESULTS

### Community-Based Urine Pregnancy Test Distribution

Of the trained FCHVs with follow-up data, 80% (n = 1,199) reported that they performed UPTs during the review period, from May 2009 to March 2010. The FCHVs performed a total of 4,598 UPTs, with a mean of 3.1 tests per FCHV. Two-thirds of FCHVs performed 1 to 5 tests, while 14% performed more than 5 tests (Table 1). FCHVs who did not provide pregnancy tests reported that women did not request a test.

**Table 1.** Distribution of UPTs Performed per FCHV

**No. of UPTs**	**Frequency (No. of FCHVs)**	**Percent Frequency**
0	293	20%
1	231	15%
2	253	17%
3	204	14%
4	149	10%
5	145	10%
> 5	217	14%
**Total**	**1492**	**100%**

Abbreviations: FCHV, female community health volunteer; UPTs, urine pregnancy tests.

The mean number of UPTs provided per FCHV varied by district. In Jhapa, in the Eastern region, each FCHV provided, on average, 5 UPTs—the highest distribution numbers among all districts (Table 2). In the Central region, FCHVs in Chitwan provided more UPTs (mean = 3.9) than in Dhading (mean = 2.6). FCHVs from Kailali in the Far Western region and Surkhet in the Mid-Western region performed similarly, providing 2.6 and 2.5 UPTs per FCHV, respectively. FCHVs in Tanahu in the Western region provided the smallest mean number of UPTs (mean = 2.1).

**Table 2.** Mean Number of UPTs Performed per FCHV by District

**District**	**No. of FCHVs**	**Mean No. of UPTs per FCHV**
Jhapa	184	5.0
Chitwan	247	3.9
Dhading	107	2.6
Kailali	533	2.6
Surkhet	341	2.5
Tanahu	80	2.1
**All Districts**	**1492**	**3.1**

Abbreviations: FCHV, female community health volunteer; UPTs, urine pregnancy tests.

### Early Pregnancy Detection, Counseling, and Referral

**Figure 2.**
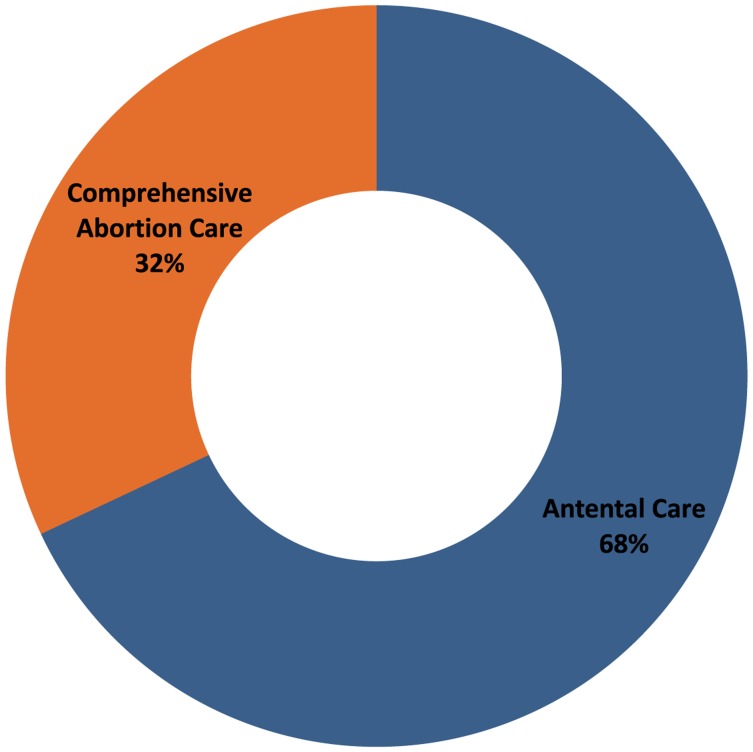
Referrals Provided to Women With Positive Pregnancy Tests (n = 2,452)

Of the 4,598 pregnancy tests performed by the FCHVs, 53% (n = 2,452) were positive and 47% (n = 2,146) were negative for pregnancy. Among women with positive pregnancy tests, FCHVs referred over two-thirds (68%) of them for ANC and about one-third (32%) for safe abortion services (Figure 2).

Health workers referred 68% of women with positive pregnancy tests for antenatal care and 32% for safe abortion.

**Figure 3.**
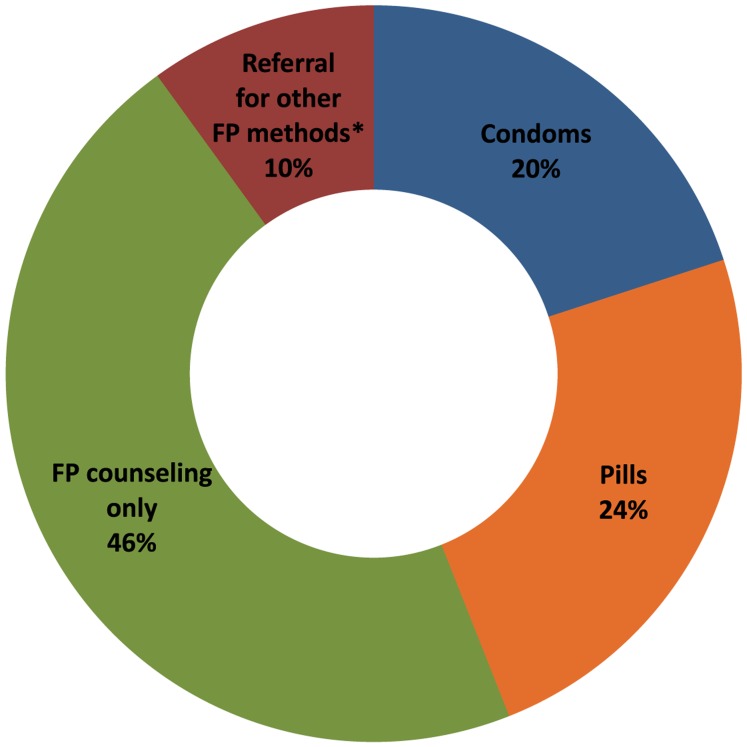
Services Provided to Women With Negative Pregnancy Tests (n = 2,146) Abbreviations: FP, family planning. * For injectables, IUDs, implants, or sterilization.

All women with negative pregnancy tests received family planning counseling. The most common family planning service provided by FCHVs was counseling only (46%), but 24% of women received oral contraceptive pills, 20% received condoms, and 10% were referred for other methods, such as injectables, IUDs, contraceptive implants, or sterilization (Figure 3).

### Perceptions of FCHVs and Other Service Providers

#### Benefits of Community-Level UPTs

FCHVs who were interviewed reported a number of positive aspects of providing UPTs at the village level:

- Tests were simple to perform, they were reliable, and the results were immediately available.
- Women in the community appreciated the low cost and local availability of the tests.
- FCHVs thought the availability of community-based early pregnancy detection increased women's privacy.
- Having pregnancy test results, combined with improved knowledge of reproductive health services from the training, enhanced their ability to provide appropriate counseling and referrals for reproductive health services.
- Women in their communities felt that early pregnancy detection and referral allowed them to make informed reproductive health decisions.

A district-level nurse discussed the value of supplying UPTs at the community level at low cost.

FCHVs perform the tests at Rs 30, whereas in district hospital they cost Rs 100. So due to this differential, the number of clients coming to hospitals for the urine pregnancy tests are decreasing nowadays.

—Nurse, District Public Health Office, Dhading

Other service providers noted an increase in FCHV referrals for ANC, family planning, and comprehensive abortion care across a variety of service sites, including public, private, and NGO clinics; hospitals; and primary health care centers.

50% of the women who come for [abortion] service are referred by FCHVs.

—Health service provider, Hospital, Tanahu

This has benefited a lot. At our centre, ANC cases are increasing. Before their [FCHVs] orientation, we use[d] to have 30–35 cases [per month] only, occasionally reaching [up] to 40–60. Nowadays, we are doing 100–150 ANC check-ups in a month. On average, 1–2 [abortion] cases are referred in a month by FCHVs.

—Health service provider, Primary Health Care Center, Dhading

#### Usefulness of IEC Materials

FCHVs reported using the pictorial IEC flip charts with women both one-on-one and in group settings, such as during Mothers Group meetings. The FCHVs found the flip charts to be an effective tool for educating women, particularly women who were uninformed about safe abortion services.

## DISCUSSION

This study found that trained FCHVs were interested and successful in providing low-cost UPTs in their communities, and pregnancy status information improved their ability to counsel and refer women for appropriate reproductive health services, including antenatal care, family planning, and comprehensive abortion care.

Among women with negative pregnancy tests, family planning counseling alone was the most common service provided (46%). More research is needed to understand why nearly half of women suspecting pregnancy but having negative pregnancy tests did not receive family planning methods or referral for methods. One possible explanation is that at least some of these women wanted to become pregnant (the record sheets did not collect information about reproductive intentions). However, it is also possible that FCHVs provided counseling alone when they did not have an adequate supply of family planning methods to distribute, or if women refused contraceptives due to fear of side effects or other health concerns.

Among women with positive pregnancy tests, FCHVs referred 68% of them for ANC and 32% for safe abortion services. Among the women referred for abortion, we do not know the proportion who actually sought CAC services. When women arrive at a certified CAC facility seeking abortion services, they receive additional counseling on options including ANC or surgical or medical abortion. After their procedures, women also receive counseling on family planning with a focus on long-acting reversible contraceptive methods. Overall, the sizable proportion of women referred for abortion services suggests that unwanted pregnancy is common, and FCHVs should focus on counseling women in their communities about family planning and providing contraceptives, including referral for long-acting and permanent methods, to prevent unwanted pregnancy.

Mobilizing a large number of FCHVs with the materials, supplies, and skills required for early pregnancy detection, reproductive health counseling, and referral seems to be a promising approach to improving access to reproductive health services at the community level in Nepal. FCHVs who were interviewed reported that women in their communities appreciated the low cost, local availability, and privacy afforded by community-based early pregnancy detection services. This evaluation was not able to link use of UPTs and referrals for services with an actual increase in uptake of services. However, we hypothesize that if FCHVs have information about available services, IEC tools to share that information, and UPTs to identify pregnancy early, and if FCHVs are providing UPTs and referring women for services, then women will have the information they need to access timely reproductive health services.

The majority of FCHVs (80%) provided UPTs. However, a sizable 20% did not provide tests during the 8-month follow-up period, primarily because they said that women did not request tests. Although it is possible that women in some communities were not interested in using UPTs, it is more likely that this finding reflects the involvement of the FCHV in her community and the proximity of the community to a health center. In communities close to a health center, women may go directly to the facility when they suspect pregnancy rather than accessing these services through the FCHV.

### Program Challenges and Lessons Learned

Program implementation challenges need to be addressed to realize the full potential of the FCHV approach, particularly related to training, resupply of UPTs, and referral.

#### Training

Training was conducted at the VDC level, but some FCHVs were unable to attend the training due to other responsibilities such as household work, personal issues, other training events, or commitment to planned Mothers Group activities. Since only 1 training was held in each VDC, FCHVs who were not available that day were not trained.

It was also difficult to secure training facilities and coordinate dates for the workshops due to concurrent programs hosted by district health offices. Trainers also noted delays and omissions in communicating training information and schedules to FCHVs, particularly at the VDC level. To improve communication, the TOT workshops were expanded to include providers at the sub-health posts, which improved coordination at the VDC level.

This pilot focused on training FCHVs working in the catchment areas for sites providing safe abortion services, which accounted for 43% of the FCHVs in these districts. Based on the findings from this pilot, training has been expanded to cover all FCHVs working in these 6 districts. As of January 2013, the program has been implemented in 19 of the 75 districts in Nepal, and Ipas intends to continue working in coordination with the Ministry of Health to scale up this program to other districts.

#### Resupply of UPTs

As discussed in the intervention description, FCHVs were asked to resupply their pregnancy test kits through local medical shops. However, this did not work well due to stockouts and to the high cost of UPTs in these shops (Rs. 50–60) when FCHVs only charged up to Rs. 50 for the resupplied kits.

For this program to be sustainable, a reliable and affordable mechanism had to be developed to resupply pregnancy test kits. Based on lessons learned during the pilot, the program contracted a distributor in Kathmandu to provide FCHVs with UPTs for Rs. 11 per test. Currently, the distributor is supplying UPTs to public health nurses at the district level, and the health post in-charges obtain their VDC's supply of UPTs from the public health nurse when they travel to the district-level health facility for their monthly meeting. The health post in-charges then provide the UPTs to the FCHVs at the VDC level. FCHVs are able to purchase the UPTs when they resupply their family planning commodities at the VDC level, which seems to be working well.

Reliable and affordable sources to resupply health workers with pregnancy tests need to be available to sustain the approach.

#### Referral

The referral card system had limited success. The FCHVs found the cards difficult to understand and to complete. Instead of providing referral cards, some of the FCHVs accompanied women to their health care provider appointments.

Based on these challenges, the program no longer uses referral cards to measure FCHV service provision. Instead, FCHVs are currently self-reporting the number of referrals they make for ANC, family planning, and safe abortion services through their monthly meetings with the medical officers in-charge. Indicators on referral for abortion services have been built into the broader health management information system (HMIS) that already monitored ANC and family planning services provided by FCHVs.

Frequent staff turnover and transfer of service providers also adversely affected the referral component of the program, which necessarily depends on the availability of reproductive health services.

### Study Limitations

The findings of this study should be viewed in light of several limitations. Around 11% of the FCHVs who were trained missed the 8-month review meeting where the assessments for this evaluation took place. The results presented here may be an overestimation of the success of the program if the FCHVs who did not attend the review meeting were less likely to implement the intervention than those who did attend.

In addition, the number of referral cards each FCHV brought to the review meeting may not have represented the true number of women served. Many FCHVs reported that they did not always provide a referral card when women came to them for help due to the informal nature of the visits and the difficulty associated with filling out the cards. Some FCHVs forgot to bring their cards to the review meeting, and instead provided an estimate for the number of women served.

Furthermore, no data were collected on the care that women received once they arrived at the referral facility. As a result, we do not know how many women who were referred actually attended a facility. We also do not know whether the result of the pregnancy test was accepted by the provider at the facility or whether women were retested. However, the purpose of this intervention was to provide women with the information they need to make timely reproductive health decisions; it was not intended to replace the care provided in health facilities.

Additionally, this study relies on data self-reported by the FCHVs, which are subject to recall and social desirability biases. Social desirability bias is of particular concern for the data from interviews about the IEC materials, as the FCHVs may have felt compelled to give positive feedback. In addition, data from the interviews were not collected systematically and may not be representative of the participants' views.

Finally, 3 of the districts included in this study were classified as hilly and 3 as flat. Including one of Nepal's mountainous districts would have provided more information relevant for scale up across all of Nepal's 75 districts.

## CONCLUSIONS

The results of this evaluation suggest that community health workers such as FCHVs are a promising channel for early pregnancy detection and referral to reproductive health services in low-resource settings. After a 2-day training on use of UPTs and referral for ANC, safe abortion, and family planning services, FCHVs provided UPTs to women in their communities and used the results of the pregnancy tests to refer women to appropriate services. FCHVs welcomed this addition to their existing menu of services, and they reported that providing this service made women feel empowered to make informed reproductive health decisions.

Health workers reported that early pregnancy detection empowered women to make timely reproductive health decisions.

Further research is needed to understand the full potential of this approach to improve access to reproductive health services at the community level. More rigorous evaluations are needed that link the use of UPTs and FCHV referrals with actual service delivery. Ideally, future studies would use an experimental design to compare changes in the proportion of services provided based on FCHV referrals in intervention districts compared with control districts with FCHVs who have not received this training. Key outcomes to assess would be the gestational age upon initiation of ANC and upon seeking safe abortion services.
